# The influence of CeF_3_ on radiation hardness and luminescence properties of Gd_2_O_3_–B_2_O_3_ glass scintillator

**DOI:** 10.1038/s41598-022-14833-3

**Published:** 2022-06-30

**Authors:** E. Kaewnuam, N. Wantana, Y. Ruangtaweep, M. Cadatal-Raduban, K. Yamanoi, H. J. Kim, P. Kidkhunthod, J. Kaewkhao

**Affiliations:** 1 Physics Program, Faculty of Science and Technology, Muban Chombueng Rajabhat University, Ratchaburi, 70150 Thailand; 2 Physics Program, Faculty of Science and Technology, Nakhon Pathom Rajabhat University, Nakhon Pathom, 73000 Thailand; 3 Center of Excellence in Glass Technology and Materials Science (CEGM), Nakhon Pathom Rajabhat University, Nakhon Pathom, 73000 Thailand; 4 Centre for Theoretical Chemistry and Physics, School of Natural Sciences, Massey University, Albany, Auckland, 0632 New Zealand; 5 Institute of Laser Engineering, Osaka University, 2-6 Yamadaoka, Suita, Osaka 565-0871 Japan; 6 Department of Physics, Kyungpook National University, Daegu, 41566 South Korea; 7 Synchrotron Light Research Institute (Public Organization), 111 University Avenue, Muang District, Nakhon Ratchasama, 30000 Thailand

**Keywords:** Condensed-matter physics, Glasses, Optics and photonics

## Abstract

The effect of CeF_3_ concentration and γ-irradiation on the physical, optical and luminescence properties of Gd_2_O_3_–B_2_O_3_–CeF_3_ glasses were studied in this work. Before irradiation, the addition of CeF_3_ in glass degraded the network connectivity observed from FTIR and possibly created the non-bridging oxygen (NBO) in glass structure. This NBO caused the reduction of Ce^3+^/Ce^4+^ ratio in XANES, the red-shift in transmission spectra and the raise of refractive index with addition of CeF_3_ content. Such red-shift also was influenced by 4f–5d transition of Ce^3+^ dopant. This ion generated the strongest photoluminescence (PL) and radioluminescence (RL) in 0.3 mol% CeF_3_-doped glass with nanoseconds decay time. The irradiation with γ-rays damaged the glass structure, broke the chemical bonds, and created color center in the glass network. The non-bridging oxygen hole center (NBOHC), that absorbed photons in the visible light region, caused the darkening, color change and increment of refractive index. These irradiation effects on glass were mitigated by the addition of CeF_3_ that the electron donation of Ce^3+^ decreased the number of NBOHC. The Ce^3+^/Ce^4+^ ratio in most glasses after irradiation then reduced compared to them before irradiation, resulting to the decrease in PL and RL intensity. Our results confirm that CeF_3_ can enhance the radiation hardness of glass and the 0.3 mol% CeF_3_-doped glass is a promising glass scintillator.

## Introduction

Single crystal scintillators are used in various applications such as medical imaging, non-destructive inspection, nuclear or high energy physics, environmental monitoring and geological exploration. In radiation detectors, single crystals offer the advantage of having high light yields and fast response times^1,2^. However, single crystal growth is an expensive and slow process; and single crystals can only be produced with limited shapes and sizes. On the other hand, glasses with various shapes and sizes are cheaper and faster to fabricate. Recently, the glass scintillators have been developed and several works have shown sufficient high light yields and fast decay times for practical applications^3–5^, including the interaction of radiation with glass and their shielding properties^6–17^. Investigation of novel glasses for radiation detection is therefore emerging, with particular focus on understanding the irradiation effects and improving the radiation hardness^18^. The radiation hardness is the resistant of material that its properties was not changed or distorted by irradiation.

Gd_2_O_3_–B_2_O_3_-based glasses are suitable scintillators owing to their radiation interaction. The ^10^B boron isotope possesses a high capture cross-section for thermal neutrons, making it a suitable neutron detector^19–21^. Additionally, B_2_O_3_ host glass is highly transparent, with good physical and chemical properties that meet the requirements for a scintillator^19,22,23^. The high phonon energy of borate glass decreases its luminescence efficiency^24^, but this can be mitigated by adding a heavy metal oxide, such as Gd_2_O_3_, into the glass^25,26^. For γ-rays and X-rays detection, the addition of Gd increases the glass density and effective atomic number which improves the interaction between glass and such incoming radiation^27–29^ and the Gd^3+^ ion can efficiently transfer the energy to luminescence centers such as lanthanide ions (Ln^3+^)^20,30,31^. In case of neutron detection, the ^155^Gd and ^157^Gd isotopes own a high capture cross-section for thermal neutrons^20,27,32^. However, there is low energy γ-rays emitted from Gd under neutron irradiation, and distinguish this γ-rays from background γ-rays is technically impossible in the pulse height or spectroscopy-based techniques. Therefore, there is no one uses Gd-based scintillators for actual applications except for some special applications. If consider in the common pulse shape discrimination it may be possible to use^33^. Therefore, the Gd_2_O_3_–B_2_O_3_-based glasses is very attractive for γ-rays and X-rays scintillator, but it has a difficulty for using in neutron detection.

Among the Ln^3+^ ions, trivalent cerium (Ce^3+^) is the most favored luminescence center for scintillator applications because the 5d–4f dipole allowed transition in Ce^3+^ results to a bright luminescence emission with nanoseconds decay time^34–36^. Previous works have investigated the Ce^3+^-doped Gd_2_O_3_–B_2_O_3_-based glasses such as Ce^3+^: Li_2_O_3_–Bi_2_O_3_–Gd_2_O_3_–B_2_O_3_^37^, Ce^3+^: Gd_2_O_3_–CaO–SiO_2_–B_2_O_3_^38^, Ce^3+^: Li_2_O–Gd_2_O_3_–BaO–B_2_O_3_^39^, Ce^3+^-Dy^3+^: CaCO_3_–ZnO–Gd_2_O_3_–B_2_O_3_^40^, and Ce^3+^: Gd_2_O_3_–B_2_O_3_^41^. In particular, our work on the xCeF_3_-doped pure binary 27.5Gd_2_O_3_–(72.5 − x)B_2_O_3_ (Ce:GB)^41^ demonstrated the significant progress in binary glass preparation as the glass sample was successfully synthesized without adding any glass modifier compound to help in glass melting process. Our technique has the advantage of excluding unnecessary oxide components which possibly degrade the color, optical and luminescence properties of the glass. Consequently, the Ce:GB glass exhibited characteristics that make it a promising glass scintillator.

In order to fully capitalize on the potential of Ce:GB and other glasses as scintillators, in-depth investigation about the effects of irradiation on the glass’ properties, especially on its luminescence properties and radiation-hardness, are necessary for further study. Consequently, the Ce:GB glasses were irradiated by gamma-rays (γ-rays) and the effect of this irradiation on glass properties were invein binary glass preparation as the glassstigated in this present work.

## Methods

Ce:GB glasses with 27.5Gd_2_O_3_-(72.5-x)B_2_O_3_-xCeF_3_ composition were synthesized by a melt-quenching technique. The glass samples, 0CeGB, 0.05CeGB, 0.1CeGB, 0.3CeGB, 0.5CeGB, 1.0CeGB and 1.5CeGB contain different CeF_3_ concentrations, with x being 0.00, 0.05, 0.10, 0.30, 0.50, 1.00 and 1.50 mol%, respectively. Details of the raw chemicals and the glass preparation procedure are stated in our previous work^41^. The Ce:GB glasses were irradiated with γ-rays carrying 1.17 and 1.33 MeV energies from a cobalt-60 (^60^Co) source. The ^60^Co source was calibrated with water and had a dose rate of 36.82 Gy/h at a distance of 1.0 m. The samples were placed at approximately 10 cm away from the radiation source for 6 h. The irradiation was performed at room temperature and in ambient atmosphere. The estimated irradiation dose rate and total dose on samples are 0.57 kGy/hour and 3.44 kGy, respectively. The oxidation state of Ce ion dopant in glasses were monitored by X-ray absorption near edge structure (XANES) spectroscopy at the Synchrotron Light Research Institute (SLRI), Thailand. The glasses densities (ρ) were determined using a 4-digit microbalance (AND, HR-200) and the Archimedes’ method^41^ with deionized water as an immersion liquid. The molar volumes of the glasses (V_M_) were calculated using the relation: V_M_ = M_T_/ρ. The refractive indices (n) of the glasses were measured by Abbe refractometer (Atago, DR-M2/M4) using the D-line (589 nm) source and 1-bromonapthalene as the contact liquid. Fourier-transform infrared (FTIR) spectra were recorded using an FTIR spectrometer (Agilent, Cary 630). An Ultraviolet–Visible–near infrared (UV–VIS–NIR) spectrophotometer (Shimadzu, UV-3600) was used to measure the transmittance spectra. The photoluminescence (PL) spectra of glasses were monitored by a spectrofluorophotometer (Agilent, Cary Eclipse) with xenon lamp as a light source. The PL decay profiles were obtained using the third harmonics (3ω, 290 nm) of a Ti:sapphire laser. The decay times were measured using a 25 cm focal length spectrograph which was fitted with a 600 grooves mm^−1^ grating that was coupled to a Hamamatsu C1587 streak camera unit and a charge-coupled device (CCD) camera. For the X-ray induced optical luminescence or radioluminescence (RL) spectra, the glass samples were excited by X-rays from a Cu target generator (Inel, XRG3D-E) with 50 kV and 30 mA power. The RL emission signal was detected by an optical fiber and a spectrometer (Ocean Optics, QE65 Pro).

## Results

### The glass appearance, density and molar volume

**Figure 1:**
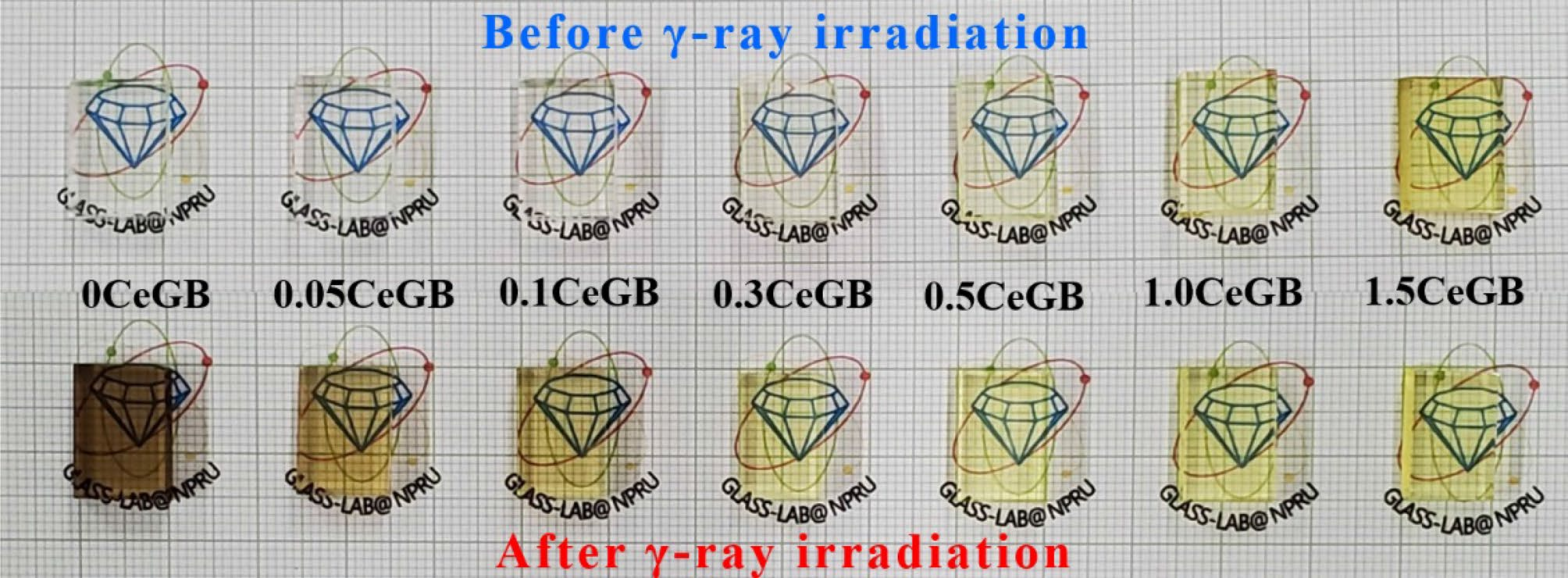
The Ce:GB glasses before and after γ-irradiation.

Photographs showing the physical appearance of Ce:GB glasses before and after γ-ray irradiation are represented in Fig. 1. Before irradiation, the CeF_3_-free glass (0CeGB) was highly transparent and colorless; while the color of the CeF_3_-doped glasses (0.05CeGB, 0.1CeGB, 0.3CeGB, 0.5CeGB, 1.0CeGB and 1.5CeGB) became more greenish yellow as the amount of CeF_3_ increased. After irradiation, the 0CeGB glass was dramatically darkened and least transparent, indicating that there was significant damage from the γ-rays. On the other hand, the irradiated CeF_3_-doped glasses was less darkened and hence more transparent than the 0CeGB glass. A greenish yellowing in the glasses can be observed which the 1.5CeGB glass visually exhibited a similar level of transparency and color tone before and after irradiation.

Table 1 shows the density (*ρ*) and molar volume (*V*_*M*_) of all Ce:GB glasses before and after γ-irradiation. The density of Ce:GB glasses were quite high in a range of 4.09–4.16 g/cm^3^, which are suitable for radiation detection^28^. The CeF_3_ concentration and irradiation did not seem to significantly affect the density and molar volume of glasses. Generally, the density of glass decreases if glass is irradiated by huge γ-rays that ejects the anions in structure. However, that density change is very small which requires high accurate measuring system to observe and the irradiation dose using in this work was not high. The increment of density after irradiation in 0.05CeGB, 0.1CeGB and 0.5CeGB glass were lower than 1%, so they could be the typical errors from measurement.

**Table 1:** Density (*ρ*) and molar volume (*V*_*M*_) of the Ce:GB glasses before^41^ and after γ-irradiation.

Glass	CeF_3_ content (mol%)	*ρ* (g/cm^3^)			*V*_*M*_ (cm^3^/mol)		
		Before	After	%Δρ	Before	After	%ΔV_M_
0CeGB	0.00	4.09	4.09	0.00	36.68	36.72	+ 0.11
0.05CeGB	0.05	4.12	4.13	+ 0.24	36.43	36.35	− 0.22
0.1CeGB	0.10	4.15	4.16	+ 0.24	36.17	36.11	− 0.17
0.3CeGB	0.30	4.14	4.14	0.00	36.40	36.32	− 0.22
0.5CeGB	0.50	4.14	4.15	+ 0.24	36.40	36.34	− 0.16
1.0CeGB	1.00	4.16	4.16	0.00	36.38	36.35	− 0.08
1.5CeGB	1.50	4.14	4.14	0.00	36.77	36.70	− 0.19

### The oxidation state of cerium ion in glass

**Figure 2:**
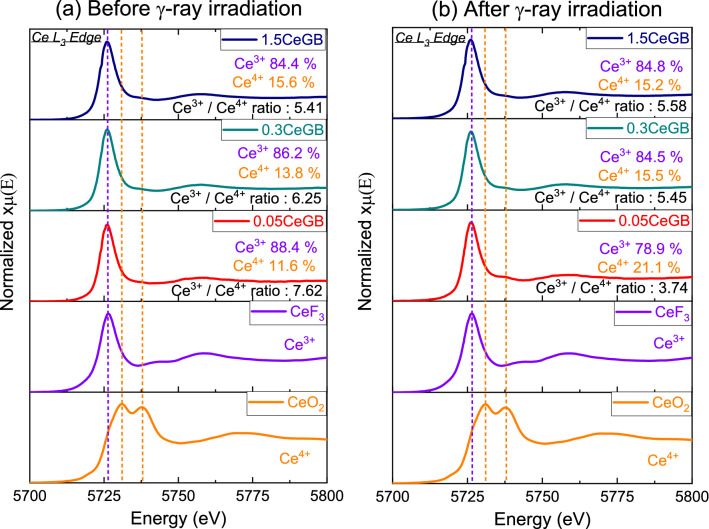
(**a**) The XANES spectra of Ce:GB glasses before and (**b**) after γ-irradiation, compared to the unirradiated CeF_3_ and CeO_2_ standard compounds.

The typical XANES spectra represent the Ce L_III_ edge of 0.05CeGB, 0.3CeGB and 1.5CeGB glasses before (Fig. 2a) and after irradiation (Fig. 2b), compared to the unirradiated standard compounds, CeF_3_ and CeO_2_. The XANES spectra show that the + 3-oxidation state of Ce^3+^ in CeF_3_ compound has a prominent absorption peak at 5727 eV, while the + 4-oxidation state of Ce^4+^ in CeO_2_ powder has obvious double peaks at 5731 eV and 5738 eV. By comparing both standard compounds, there was also a weak absorption peak of Ce^3+^ in the CeO_2_ powder. Likewise, there were weak peaks of Ce^4+^ in the CeF_3_ compound. These indicate that the cerium ions in CeF_3_ and CeO_2_ coexisted in both Ce^3+^ and Ce^4+^ states. The Ce:GB glasses in this work were doped with CeF_3_. Therefore, their XANES spectra mimiced the spectrum of CeF_3_ standard where the Ce^3+^ ion is dominant. The XANES data were evaluated using the Athena software to ascertain the quantity percentage of Ce^3+^ and Ce^4+^ ions in Ce:GB glasses. Before irradiation, the ratio of Ce^3+^/Ce^4+^ in the glasses decreased as the CeF_3_ concentration increased. The same trend was also observed in CaO–SiO_2_–B_2_O_3_–CeF_3_ and SiO_2_–Al_2_O_3_–Li_2_O–Na_2_O–K_2_O–BaO–SrO–Tb_2_O_3_–Gd_2_O_3_–CeO_2_ glass fabricated in air atmosphere by Rajaramakrishna et al.^38^ and Zu et al.^42^, respectively. On the other hand, the Ce^3+^/Ce^4+^ ratio in the glasses after irradiation increased with the increase in CeF_3_ concentration. Considering the effect of γ-irradiation, it decreased the Ce^3+^/Ce^4+^ ratio of 0.05CeGB and 0.3CeGB glasses while it slightly increased this ratio in 1.5CeGB glass.

### The glass network

**Figure 3:**
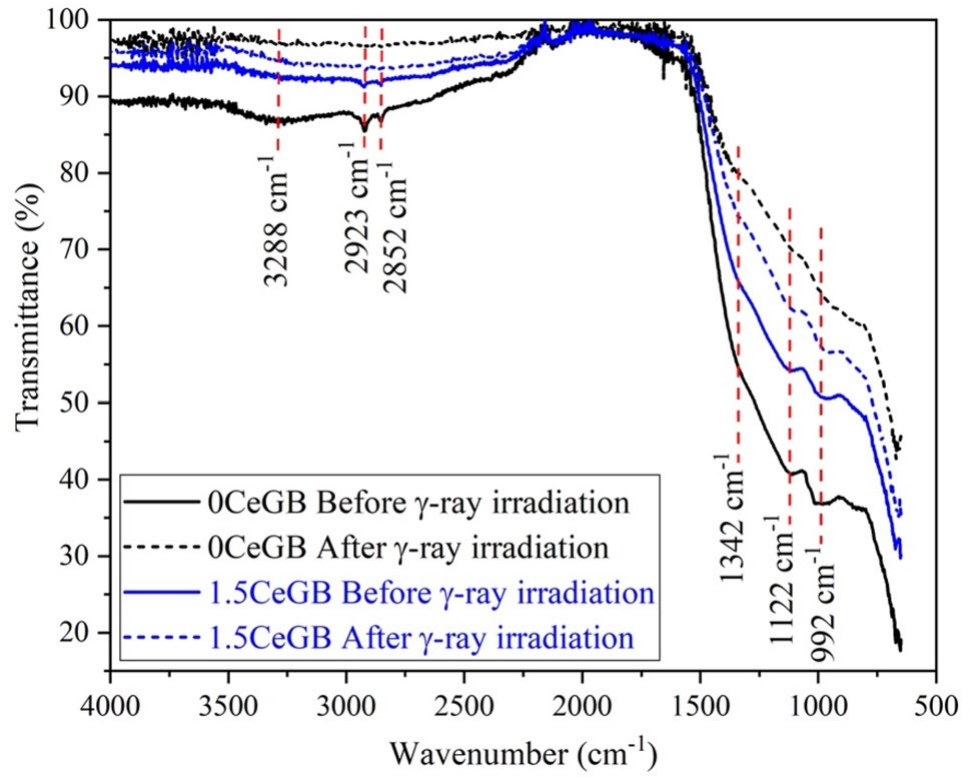
The FTIR of 0CeGB and 1.5CeGB glass before/after γ-irradiation.

Results of the FTIR measurements for the 0CeGB and 1.5CeGB glasses before and after γ-ray irradiation in Fig. 3 indicate that the borate group was the main structural unit in glass network. The infrared vibration at 992 cm^−1^ corresponded to the B–O stretching vibration of tetrahedra BO_4_ units in tri-, tetra- and pentaborate^27,43,44^. While the B–O stretching of trigonal BO_3_ and tetrahedra BO_4_ units were attributed to the vibration around 1122 cm^−1^^27,43^. The FTIR absorption around 1342 cm^−1^ was assigned to the B–O stretching vibration of the trigonal (BO_3_)^3−^ units in meta-, pyro- and orthoborates^27,43^. The vibration centered at 2923 and 2852 cm^−1^ corresponded to the O–H stretching of hydroxyl OH^−^ groups, while the broad band around 3288 cm^−1^ revealed to the vibration of OH^−^ groups and B–OH linkage^27,45^. Before γ-irradiation, the vibration strength of these BO_4_, BO_3_, OH groups and B-OH linkage in 1.5CeGB glass were weaker than 0CeGB glass, indicating that the chemical groups in CeF_3_-doped glasses have poorer connectivity compared to the undoped glass. After irradiation, the γ-rays could break some chemical bonds in the glass network, resulting to the decrease in vibration strength of those chemical complexes. The infrared absorption by such complexes then reduced which caused the increment of FTIR transmittance after irradiation. The change in vibration strength of 1.5CeGB glass due to γ-rays damage was less than that of the 0CeGB glass.

### The optical properties

**Figure 4:**
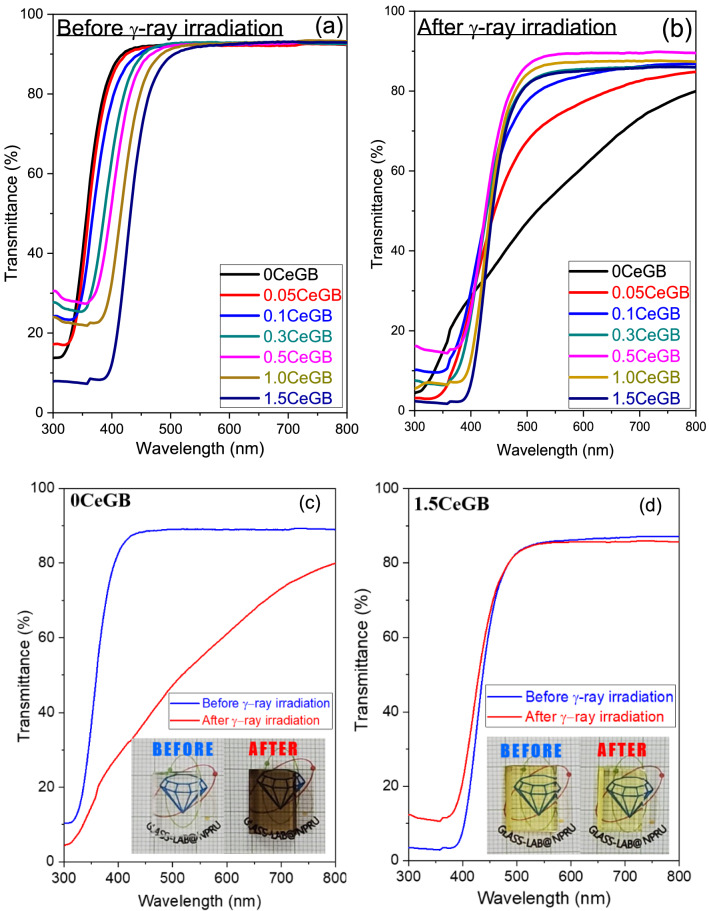
(**a**) Transmission spectra of Ce:GB glasses before γ-irradiation^41^ and (**b**) after γ-irradiation, (**c**) The comparative transmission spectra and pictures before/after γ-irradiation of 0CeGB and (**d**) 1.5CeGB glass.

The transmission spectra of the Ce:GB glasses before γ-irradiation are shown in Fig. 4a. The unirradiated 0CeGB glass exhibited strong absorption in UV region with a transmission edge wavelength around 320 nm and the transmission spectra was shifted to longer wavelength with addition of CeF_3_ content. This red-shift of glasses influenced by CeF_3_ concentration were also found in several literatures^18,34,35,38,46^. Considering on the effect of γ-rays, the 0CeGB glass after irradiation obviously absorbed photons in UV and VIS regions, as shown in Fig. 4b. The γ-rays generated the color center that increased the absorption in both regions, especially in the VIS range.

The increased absorption after irradiation is called “radiation-induced absorption” that can be considered from the change of transmittance before (*T*_0_) and after (*T*) irradiation at each wavelength by following relation^47^,

1 $$T={T}_{0}{e}^{-{\alpha }_{D}x},$$

where *α*_*D*_ is the radiation-induced absorption coefficient at each wavelength and *x* is the optical path length or thickness of sample. The *α*_*D*_ were calculated in a range of VIS, 400–800 nm that the typical coefficient at 477 nm (*α*_*D-477*_) and the average coefficient (*α*_*D-ave*_) were shown in Table 2. The decrease of *α*_*D*_ values represented the less effect of irradiation on glass with added CeF_3_ concentration, corresponding to the comparative transmission spectra and pictures of 0CeGB and 1.5CeGB glasses in Fig. 4c,d. The transmittance and color of 0CeGB glass changed significantly by irradiation, but they were very similar for 1.5CeGB glass.

**Table 2:** The radiation-induced absorption coefficient at 477 nm (*α*_*D-477*_) and its average value in a range of 400–800 nm (*α*_*D-ave*_); and the CIELAB parameters (L^*^, a^*^ and b^*^) of the Ce:GB glasses before/after γ-irradiation.

Glass	*α*_*D*_ (cm^−1^)		CIELAB parameters									
			L*			a*			b*			*∆E*_ab_*
	*α*_*D-477*_	*α*_*D-ave*_	Before	After	∆L*	Before	After	∆a*	Before	After	∆b*	
0CeGB	2.42	1.48	95.58	79.22	− 16.36	− 0.30	1.99	2.29	0.49	18.16	17.66	24.18
0.05CeGB	1.18	0.78	95.61	88.66	− 6.95	− 0.38	− 2.70	− 2.32	0.67	17.23	16.56	18.11
0.10CeGB	0.54	0.45	94.25	92.37	− 1.88	− 0.78	− 4.15	− 3.36	1.66	14.99	13.34	13.88
0.30CeGB	0.42	0.37	95.32	93.53	− 1.80	− 1.53	− 5.55	− 4.01	3.04	14.47	11.43	12.24
0.50CeGB	0.27	0.26	96.09	95.33	− 0.75	− 2.20	− 5.87	− 3.67	4.50	14.25	9.75	10.44
1.00CeGB	0.22	0.25	95.16	94.39	− 0.77	− 4.24	− 7.02	− 2.78	9.47	16.83	7.36	7.91
1.50CeGB	0.13	0.09	94.24	93.33	− 0.92	− 7.23	− 7.48	− 0.26	17.48	18.59	1.11	1.47

The transmittance spectra were used to analyze the color parameters (L^*^, a^*^ and b^*^) of glasses using the CIE 1976 L^*^a^*^b^*^ Color Space (CIELAB)^48^ The values of CIELAB parameters are represented in Table 2. The magnitude of L^*^ represents the brightness level, the positive/negative value of a^*^ indicates the red/green approach and the positive/negative value of b^*^ implies the yellow/blue approach of specimen. In glasses before γ-irradiation, more negative value of a^*^ and more positive value of b^*^ with increasing CeF_3_ content corresponded to the change of glass color becoming more greenish yellow, as can be seen in Fig. 1. After irradiation, γ-rays decreased the L*, changed the a* to be positive and increased the a*, resulting to the dark tone color of undoped 0CeGB glass. For CeF_3_-doped glasses after irradiation, the change of L* and b^*^ tended to be less while the change of a* was fluctuating with addition of CeF_3_. To evaluate the total change of glass color that is damage from γ-rays, the color difference (*∆E*_ab_^*^) between glasses before and after irradiation were calculated by equation^48^,

2 $${\Delta E}_{ab}^{*}=\sqrt{{\left({\Delta L}^{*}\right)}^{2}+{\left({\Delta a}^{*}\right)}^{2}+{\left({\Delta b}^{*}\right)}^{2}},$$

where ∆L^*^, ∆a^*^ and ∆b^*^ is the difference values of such color parameters, before and after irradiation. The *∆E*_ab_^*^ value decreased with increment of CeF_3_ concentration corresponding to more similar color that the 1.50CeGb glass after irradiation came back to be bright and greenish yellow as same as itself before irradiation.

The refractive index (*n*) of the glasses are shown in Table 3. The value of *n* for unirradiated glasses increased as the amount of CeF_3_ increased. Considering the effect of γ-rays, the refractive index of irradiated glasses were higher than their unirradiated value. The difference in refractive index (Δ*n*) before and after irradiation progressively decreased with increment of CeF_3_ content. All *n* values were used to calculate the molar refraction (*R*_*m*_) and the molar polarizability (*α*_*m*_), respectively by following relations^49,50^,

3 $${R}_{m}=\left(\frac{{n}^{2}-1}{{n}^{2}+2}\right){V}_{M ,}$$

4 $${\alpha }_{m}=\left(\frac{3}{4\pi N}\right){R}_{m}\text{,}$$

where *N* is the Avogadro’s number. The *α*_*m*_ parameter is the net electronic polarizability in glass that indicates the response of electrons to the electric field from incoming electromagnetic wave^49,50^, D-line light in this case. The variations of *α*_*m*_ value influenced by the CeF_3_ concentration and γ-irradiation were in similar trend with the *n* value as shown in Table 3. This means the electrons in glasses were more sensed and the molecules were easily polarized to such electric field by the increment of CeF_3_ dopant and the γ-irradiation. The change of polarizability due to irradiation (Δ*α*_*m*_) was smaller with adding CeF_3_ concentration.

**Table 3:** The refractive index (*n*), molar refraction (*R*_*m*_) and molar polarizability (*α*_*m*_) of the Ce:GB glasses before and after γ-irradiation.

*n*			*R*_*m*_ (cm^3^/mol)		*α*_*m*_ (× 10^–24^ cm^3^)		
Before	After	Δ*n*	Before	After	Before	After	Δ*α*_*m*_
1.4718	1.6915	+ 0.2198	10.2687	14.0580	4.0749	5.5786	+ 1.5037
1.5394	1.6892	+ 0.1497	11.4200	13.9125	4.5318	5.5208	+ 0.9891
1.5411	1.6895	+ 0.1484	11.3688	13.7931	4.5114	5.4735	+ 0.9620
1.5756	1.6916	+ 0.1160	12.0395	13.9089	4.7776	5.5194	+ 0.7419
1.6120	1.6961	+ 0.0841	12.6522	13.9834	5.0207	5.5490	+ 0.5283
1.6331	1.6945	+ 0.0615	12.9955	13.9655	5.1569	5.5418	+ 0.3849
1.6728	1.6915	+ 0.0187	13.7802	14.0527	5.4683	5.5765	+ 0.1081

**Figure 5:**
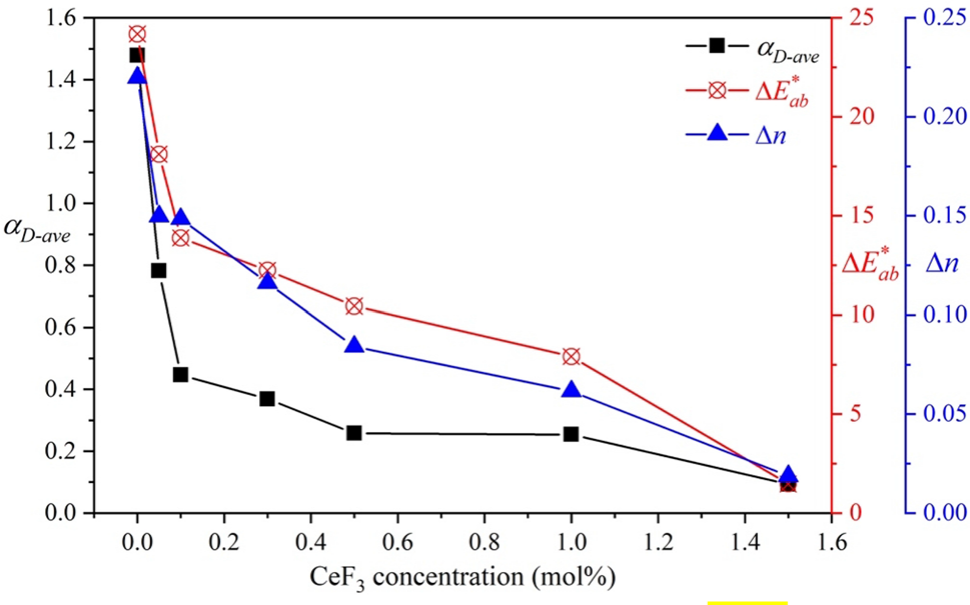
The average radiation-induced absorption coefficient (*α*_*D-ave*_), color difference (*∆E*_ab_^*^) and refractive index difference (Δ*n*) of Ce:GB glasses.

The *α*_*D-ave*_, *∆E*_ab_^*^ and Δ*n* value as a function of CeF_3_ concentration were plotted cooperatively in Fig. 5 which those parameters owned the similar behavior on variation of CeF_3_ content. This represents the ability of CeF_3_ that enhanced the radiation hardness on glass optical properties.

### The photoluminescence spectra and decay curves

**Figure 6:**
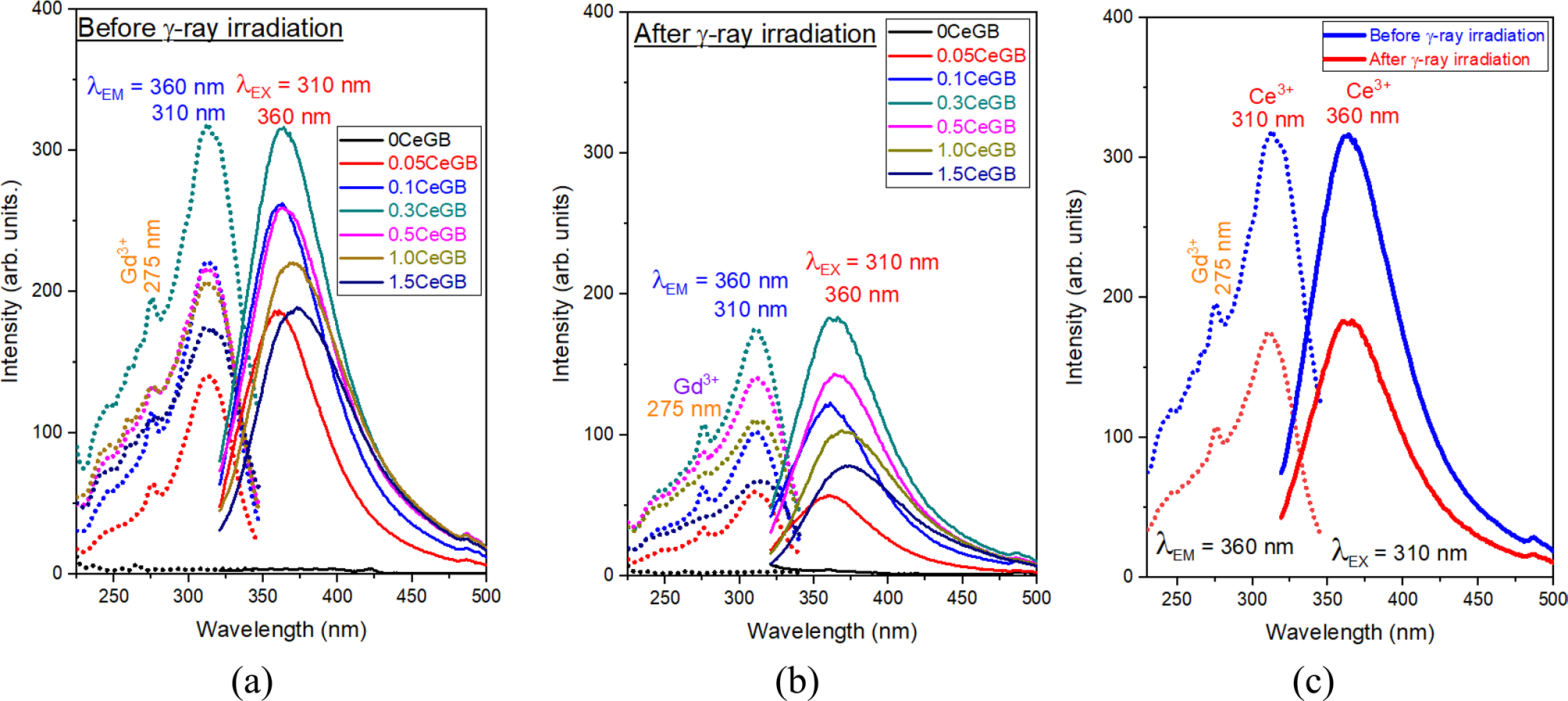
(**a**) The PL emission and excitation spectra under Ce^3+^ excitation of Ce:GB glasses before γ-irradiation and (**b**) after γ-irradiation, (**c**) the comparative PL spectra before and after γ-irradiation of 0.3CeGB glass.

**Figure 7:**
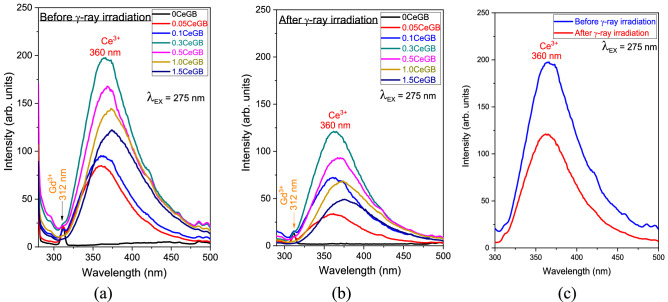
(**a**) The PL emission spectra under Gd^3+^ excitation of Ce:GB glasses before γ-irradiation^41^ and (**b**) after γ-irradiation, (**c**) the comparative PL emission spectra before and after γ-irradiation of 0.3CeGB glass.

**Figure 8:**
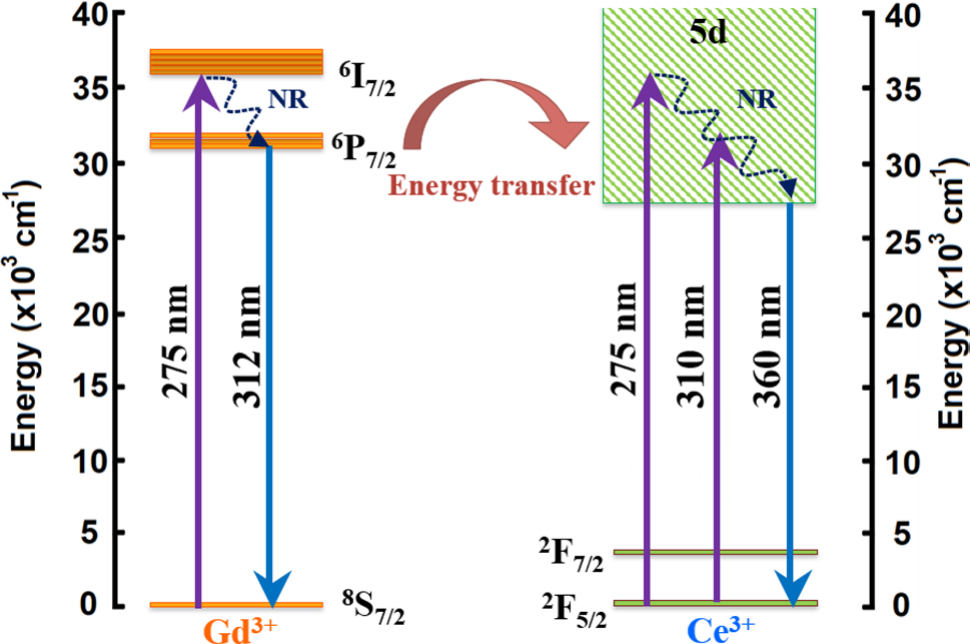
The possible energy level diagram for PL spectra of Ce:GB glasses.

The PL emission (solid line) and excitation (dash line) spectra under direct Ce^3+^ excitation of glasses before irradiation are shown in Fig. 6a. The luminescence intensity increased with increasing CeF_3_ concentration in the range of 0.00–0.30 mol%. The intensity decreased for CeF_3_ amounts larger than 0.30 mol% due to concentration quenching. The UV with 310 nm wavelength directly excited to the Ce^3+^ and promoted this ion from the ground 4f (^2^F_5/2_) to the excited 5d state. The Ce^3+^ then relaxed to the lowest vibrational 5d state via a non-radiative relaxation (NR) process, followed by the 5d → 4f (^2^F_5/2_) transition where a photon with 360 nm wavelength was emitted^34,38,40^. After irradiation, the PL spectra in Fig. 6b shows a similar peak position as the spectra before irradiation. Concentration quenching was also observed when the CeF_3_ doping concentration was more than 0.30 mol%. However, the luminescence intensity of the irradiated glasses decreased compared to the unirradiated ones. A clear evidence of intensity degradation is represented in the comparative spectra of 0.3CeGB glass in Fig. 6c. Moreover, an excitation peak around 275 nm of Gd^3+^ was also found and it overlapped on the left side of the Ce^3+^ excitation peak in those Fig. 6. The emission spectra of glasses under Gd^3+^ excitation were then studied and shown in Fig. 7a–c. The peak position, the influence of CeF_3_ concentration and γ-irradiation on emission intensity were similar with the spectra under direct Ce^3+^ excitation. Additionally, a small peak of Gd^3+^ emission under ^6^P_7/2_ → ^8^S_7/2_ transition was found at 312 nm wavelength. The strength of the Gd^3+^ emission peak was weakened with increasing CeF_3_ concentration because the excitation energy of Gd^3+^ was more transferred to Ce^3+^. The mechanism is as follows: the UV excitation with 275 nm excited the Gd^3+^ from ^8^S_7/2_ to ^6^I_7/2_ state. NR then took Gd^3+^ down to ^6^P_7/2_ level which was the intersection for next two separate routes. The first one was the ^6^P_7/2_ → ^8^S_7/2_ transition where Gd^3+^ emitted the photon with 312 nm. For the second route, the energy transferred from ^6^P_7/2_ state of Gd^3+^ to 5d state of Ce^3+^. After that, the 5d → 4f (^2^F_5/2_) transition of Ce^3+^ emitted the photon with 360 nm^34,38,40^. Furthermore, there was a probability that the excitation with 275 nm also directly excited to Ce^3+^ because its energy range of 5d state in glass is wide and overlaps with the ^6^I_7/2_ state of Gd^3+^. This appeared as the overlapping between the excitation peak of Gd^3+^ and Ce^3+^ in the PL spectra. The possible mechanisms about the energy transition of Ce^3+^ and Gd^3+^ in the PL spectra are represented in Fig. 8.

**Figure 9:**
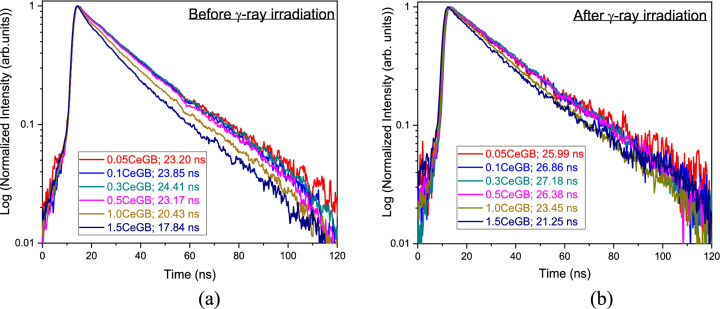
(**a**) The decay curves under 290 nm excitation of Ce:GB glasses before γ-ray irradiation and (**b**) after γ-irradiation.

The decay curves of Ce:GB glasses under 290 nm excitation before and after irradiation are shown in Fig. 9a,b, respectively. All decay curves were fitted well with a single exponential function. The decay time values before irradiation were in the range of 17.84–24.41 ns and their values after irradiation were between 21.25 and 27.18 ns. These short decay times in the order of tens of nanoseconds are the signature of Ce^3+^ luminescence under the 5d → 4f transition^34,39,46^. The decay time values increased with increasing CeF_3_ content in the range of 0.00–0.30 mol%, while they decreased for CeF_3_ concentration ranging from 0.30 to 1.50 mol%. This variation of decay time was similar with the change of PL intensity influenced by CeF_3_ amount. Moreover, it was found that the γ-irradiation caused an increment of decay time value in each glass.

### The radioluminescence spectra and decay curves

**Figure 10:**
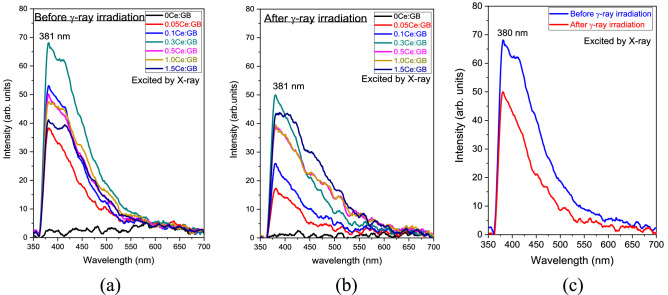
(**a**) The RL spectra of Ce:GB glasses before γ-irradiation^41^ and (**b**) after γ-ray irradiation, (**c**) the comparative RL spectrum before and after γ-ray irradiation of 0.3CeGB glass.

The RL spectra before and after γ-irradiation in Fig. 10 show a strong emission from Ce^3+^ around 381 nm wavelength. The incoming X-rays could initially interact to the glass host. The X-rays energy then transferred to luminescence center (Gd^3+^ or Ce^3+^). After that, Gd^3+^ could emit 312 nm luminescence but could not be detected in this experiment because of lower limit detection of spectrometer. There is also another possibility of Gd^3+^ transferred the energy to Ce^3+^ for luminescence under the 5d → 4f transition, similar process with the PL spectra. Additionally, the 5d–4f transition of Ce^3+^ could be occurred from this scintillation process. The RL intensity of glasses tended to quench for CeF_3_ concentrations that was greater than 0.30 mol%, like what was observed in the PL spectra. The γ-irradiation degraded the RL intensity of each glass which can be clearly observed in the comparative RL spectra of 0.3CeGB glass in Fig. 10c. The RL intensity of 0.3CeGB glass after irradiation decreased by 35% compared to its pre-irradiation intensity.

## Discussion

### Before γ-irradiation

In this part, only the influence of CeF_3_ concentration on glasses before γ-irradiation are discussed and some explanations will be used continuously in the next subsection. The CeF_3_ dopant possibly acted as a glass modifier that created non-bridging oxygen (NBO) and disrupted the connectivity of borate groups in the Gd_2_O_3_-B_2_O_3_ glass network. Consequently, the FTIR vibration strengths of those borate complexes in 1.5CeGB were weaker than in 0CeGB glass. The vibration of OH^-^ groups in FTIR were also reduced by CeF_3_ increment due to the reaction with F^-^ ion as followed, 2OH^−^ + 2F^−^ → 2HF +in binary glass preparation as the glass O_2_^41,51^. Since the transmission edge of host 0CeGB glass was overlapping to the 4f.-5d transition of Ce^3+^, the transmission spectra of CeF_3_-doped glasses then were red-shifted by more influence of this transition via increasing CeF_3_ dopant. This red-shift caused the change of CIELAB parameters in Table 2 and the observed change of color becoming more greenish yellow in Fig. 1. Additionally, there were reported that the addition of NBO could increase the glass optical basicity and consequently affected to the red-shift in absorption–transmittance spectra^34,52–54^. The electrons at NBO sites in glass are less tightly bound and can be easily oscillated by the electric field from incoming light, compared to electrons at bridging oxygen sites^34,52^. The polarizability of glass then increased by following number of NBO with addition of CeF_3_ content. Since the light was more sensed by the electrons, this light-electron interaction slowed down the speed of light (*v*) in glass resulting to the increment of refractive index by *n* = *c*/*v* relation with added CeF_3_ concentration.

The electronic configuration of Ce^3+^ ion is 4f^1^, which means that it has only one electron in the 4f shell to lose in order to have a more stable empty state. Therefore, Ce^3+^ can change to Ce^4+^ by losing one of its 4f. electron through the direct ionization, thermal ionization, or donation to hole by the process: Ce^3+^  + hole → Ce^4+^. On the other hand, a Ce^4+^ ion can accept an electron to form Ce^3+^ via the reaction: Ce^4+^  + electron → Ce^3+^^38,41^. This causes the coexistence of Ce^3+^ and Ce^4+^ ion in cerium doped materials, such as our glasses in this work. Since the electrons at NBO sites are less tightly bound, these electrons density could distribute and affect to the behavior of an 4f electron of Ce^3+^. The electrostatic pull between Ce^3+^ nucleus and its 4f orbital was weaken by such negative charges from NBO^41,55,56^. This increased the probability of Ce^3+^/Ce^4+^ ratio reduction with increasing of CeF_3_ content as observed in the XANES spectra. Concentration quenching was found in both the PL and RL spectra of glasses doped with CeF_3_ higher than 0.3 mol%. Quenching is due to the re-absorption of photons that are emitted by closely nearby Ce^3+^ neighbors. The shorter distance between Ce^3+^ ions and the dense ion distribution in glass provide this quenching effect, which also led to the reduction of decay time for glasses with more than 0.3 mol% of CeF_3_.

### After γ-irradiation

Considering the 0CeGB glass after irradiation, γ-rays could break some B–O–B and B–O–H linkages by following Eqs. (5) and (6), respectively.

5 $$\equiv \mathrm{B}{-}\mathrm{O}{-}\mathrm{B}\equiv \stackrel{\mathrm{irradiation}}{\to } \equiv \mathrm{B}\cdot \cdot \mathrm{O}-\mathrm{B}\equiv ,$$

6 $$\equiv \mathrm{B}{-}\mathrm{OH }\stackrel{\mathrm{irradiation}}{\to } \equiv \mathrm{B}{-}\mathrm{O}\cdot +\mathrm{ H}^\circ .$$

Both “⋅ O –” and “– O ⋅” are the NBO, while the “≡ B ⋅” is the deformed borate complexes^45^. Generally, the NBO and deformed borate are the charge defect which naturally pre-exist in the unirradiated metal-oxide borate glasses, also in our Gd_2_O–B_2_O_3_ system, the γ-irradiation just increased the number of these complexes. The γ-rays could also ionize the chemical composition that generated the electron and hole in glass structure^47^. This hole and electron could separate and move to trap with those charge defects in glass. Hole could be trapped by negative charge of NBO to form the non-bridging oxygen hole center (NBOHC). The electron was probably trapped by positive charge of deformed borate, becoming to the boron electron center (BEC)^45,57,58^. However, there was reported that the BEC in borate glass was unstable for temperature above 120 K and its number dramatically decreased to be negligible at about 320 K^59,60^. Therefore, the main color center in our glasses after irradiation is NBOHC. This hole center is thought to absorb the photon around 3.8 eV (326 nm) and 2.6 eV (477 nm), that’s why the darkening and color change was obviously appeared in 0CeGB glass^47,57,61,62^. This corresponded to the high value of radiation-induced absorption coefficient at 477 nm and its average value in VIS range of this glass as shown in Table 2. The radiation-induced absorption coefficients in UV range lower than 400 nm were not analyzed due to the overlapping of 4f.-5d transition from Ce^3+^ on absorption of glass host. Since the irradiation possibly destroyed the B–O–B, B–O–H linkage and OH group shown in Eqs. (5) and (6), and disrupted the glass structure by formation of NBOHC, the FTIR vibration strength of 0CeGB glass then significantly decreased after irradiation. Moreover, the charge complexes such as the NBO and NBOHC created by γ-rays raised obviously the value of polarizability and refractive index in this glass.

For CeF_3_-doped glasses after irradiation, the electron donation from Ce^3+^ to hole inhibited the hole trapping at the charge defect site such as NBO^38,47,61,63^. Consequently, the number of NBOHC was decreased and the structure of 1.5CeGB glass was more conserved from the disruption than 0CeGB glass, as shown in FTIR spectra. The NBOHC reduction with the addition of CeF_3_ content also caused a decrease of those radiation damage parameters such as the radiation-induced absorption coefficient, the color difference, the change of polarizability and refractive index. Especially in 1.5CeGB glass, these values were close to zero which represented the highest radiation hardness.

The donation of an electron from Ce^3+^ to hole caused the reduction of Ce^3+^/Ce^4+^ ratio in 0.05CeGB and 0.3CeGB glass after irradiation, observed by XANE spectra. For 1.5CeGB glass, the large amount of CeF_3_ dopant created high number of pre-existed NBO in the glass network, and there were the electrons created by irradiation those could not trap to BEC because this center was unstable as previously mentioned. Some Ce^4+^ possibly accepted an electron from NBO and unstable BEC which changed this ion back to Ce^3+^. The Ce^3+^/Ce^4+^ ratio in 1.5CeGB glass therefore slightly increased by irradiation. The PL and RL luminescence intensity of CeF_3_-doped glasses decreased after irradiation due to the reduction of Ce^3+^/Ce^4+^ ratio. Since the absorption energy of defect (NBOHC at 3.8 eV) in UV region overlapped to the 4f–5d transition of Ce^3+^ in this glass, the UV excitation energy on decay time measurement was possibly trapped by defect, resulting to longer decay time after irradiation^64^.

From all results, the 0.3CeGB glass is a promising new glass scintillator, with the highest emission intensity among the glasses studied in this work, a relatively fast nanoseconds decay time and excellent radiation hardness.

## Conclusion

Various properties of CeF_3_-doped Gd_2_O_3_-B_2_O_3_ glasses before and after γ-irradiation were comparatively investigated. XANES results show that the major and minor oxidation states of cerium ion in glasses were Ce^3+^ and Ce^4+^, respectively. Before irradiation, the analysis of glasses’ transparency, FTIR, refractive index and polarizability indicated that CeF_3_ degraded the connectivity and possibly created NBO in glass structure. This NBO caused the reduction of Ce^3+^/Ce^4+^ ratio, the red-shift in transmission spectra and the raise of refractive index with addition of CeF_3_ content. That red-shift also was influenced by 4f–5d transition of Ce^3+^ dopant. After irradiation, γ-rays damaged the glass structure, broke the chemical bond, and created color center in the borate network former. That center is NBOHC which absorbed photons in VIS region, resulting to the darkening and color change in glasses after irradiation. Moreover, the polarizability and refractive index of glasses were increased by the formation of NBO and NBOHC generated by irradiation. The addition of CeF_3_ concentration in glass relieved these irradiation effects. Due to the electron donation from Ce^3+^ to hole, number of NBOHC were annihilated. The radiation damage indicators such as the radiation-induced absorption coefficient, the color difference, the change of polarizability and refractive index then decreased in value with increasing CeF_3_ dopant. These results confirm the ability of CeF_3_ that enhances the radiation hardness of glass. The PL and RL intensity of CeF_3_-doped glasses decreased after irradiation due to the reduction of Ce^3+^/Ce^4+^ ratio via electron donation of Ce^3+^. The decay times of glasses after irradiation were longer, compared to them before irradiation because the excitation energy was possibly trapped by defect (NBOHC). The Gd_2_O_3_-B_2_O_3_ glass doped with 0.30 mol% of CeF_3_ exhibited the highest emission intensity, fast 24–27 ns decay time and owned the radiation hardness property, making it a promising new glass scintillator.
